# Functions of mountain pine beetle cytochromes P450 CYP6DJ1, CYP6BW1 and CYP6BW3 in the oxidation of pine monoterpenes and diterpene resin acids

**DOI:** 10.1371/journal.pone.0216753

**Published:** 2019-05-09

**Authors:** Christine C. Chiu, Christopher I. Keeling, Hannah M. Henderson, Joerg Bohlmann

**Affiliations:** 1 Michael Smith Laboratories, University of British Columbia, Vancouver, British Columbia, Canada; 2 Department of Botany, University of British Columbia, Vancouver, British Columbia, Canada; Ghent University, BELGIUM

## Abstract

The mountain pine beetle (MPB; *Dendroctonus ponderosae*) is a forest insect pest that attacks several different pine (*Pinus*) species in its native range of distribution in western North America. MPB are exposed for most of their life cycle to the chemical defenses of their hosts. These defenses are dominated by oleoresin secretions containing mostly various monoterpenes and diterpene resin acids (DRAs). Cytochrome P450 enzymes (P450s) of the MPB are thought to be involved in the metabolism of at least some of these defense compounds. Here we describe the cloning and characterization of three MPB P450s, CYP6DJ1, CYP6BW1 and CYP6BW3, and their functions in the oxidation of various monoterpenes and diterpene resin acids. CYP6DJ1 oxidizes the monoterpenes (+)-(4*R*)-limonene, (–)-(4*S*)-limonene and terpinolene and produces (4*R*,8*R*)-limonene-8,9-epoxide, (4*R*,8*S*)-limonene-8,9-epoxide, (4*S*,8*S*)-limonene-8,9-epoxide, (4*S*,8*R*)-limonene-8,9-epoxide, perilla alcohol and several unidentified oxidized compounds. These products of CYP6DJ1 were also identified in extracts of MPB treated with the same monoterpenes. CYP6BW1 and CYP6BW3 both oxidize the DRAs abietic acid, dehydroabietic acid, neoabietic acid, levopimaric acid, palustric acid, and isopimaric acid, producing hydroxylated and epoxidized DRAs. CYP6DJ1, CYP6BW1 and CYP6BW3 appear to contribute to the metabolism of oleoresin terpenes as part of the MPB’s ability to cope with host defenses.

## Introduction

The mountain pine beetle (MPB; *Dendroctonus ponderosae*) is a forest pest that attacks pine forests across western North America [1]. As part of its lifecycle, which is spent mostly in the phloem of its various pine (*Pinus*) hosts, the MPB is exposed to the tree’s oleoresin defenses. Oleoresin is mostly comprised of monoterpenes and diterpene resin acids (DRAs) [2]. The relationship between MPB and host terpenoids has been studied extensively, as these compounds have chemo-ecological roles not only as defense chemicals, but also as volatile signal molecules by which MPB identify suitable hosts and as MPB pheromone precursors [3]. While some monoterpenes are toxic to MPB [4], MPB and its microbial associates can detoxify some of the oleoresin metabolites [5–10]. MPB-associated fungi can utilize some of the oleoresin terpenes, specifically (+)-(4*R*)-limonene, as a carbon source [7]. Female MPB produce the aggregation pheromone *trans*-verbenol from the host monoterpene α-pinene, which is abundant in pine oleoresin [11–13].

The MPB genome contains 86 different cytochrome P450 genes [14], and three of these P450s have previously been shown to function in terpenoid metabolism or terpenoid pheromone biosynthesis in the MPB [13,15,16]. At least seven different MPB P450s are differentially expressed in organs or tissues where metabolism of monoterpenes and DRAs is likely to occur, specifically in antennae for olfaction, as well as in the alimentary canal and fat body for detoxification and pheromone formation [13,17,18]. Of these seven P450s, CYP345E2 metabolizes the monoterpenes (+)-3-carene, (–)-camphene and both enantiomers of α-pinene, β-pinene and limonene [15], and CYP6DE1 oxidizes (+)-3-carene, and both enantiomers of α-pinene, β-pinene and converted (–)-α-pinene to (–)-*trans*-verbenol, an aggregation pheromone released by female MPB [13]. Among this same set of seven genes, transcripts of *CYP6DJ1* and *CYP6BW3* were highly abundant in antennae, while *CYP6BW1* was highly abundant in the midgut [17]. *CYP6BW1* and *CYP6BW3* share 96% amino acid identity, suggesting that they originated from a gene duplication. Their expression in different tissues indicates divergent biological functions. Analysis of transcript abundance in male and female MPB at different life stages, specifically 3^rd^ instar larvae, pupae, and teneral, emerging and colonizing adults also revealed sex-specific differences in the expression of these P450s [17]. Transcript abundance of *CYP6DJ1* was significantly higher in colonizing females compared to colonizing males, while transcript abundance of *CYP6BW3* was higher in colonizing males. CYP6BW1 did not show sex-specific differences in expression.

Here, we investigated biochemical functions of CYP6DJ1, CYP6BW1 and CYP6BW3 by testing each of the three heterologously expressed P450s in a series of *in vitro* assays with ten different monoterpenes and six DRAs as substrates. The substrates were selected to include major oleoresin compounds of common MPB hosts. We compared the monoterpene oxidation products of the P450 *in vitro* assays with products formed *in vivo* by female MPB that were exposed to monoterpenes.

## Materials and methods

### Insects

MPB infested lodgepole pine (*Pinus contorta*) trees were felled near Whistler, BC, Canada (50°12’33.3”N 122°53’05.2”W) from a forest license area managed by Halray Logging Ltd in October 2015. Permission to collect infested trees were provided by Halray Logging Ltd. The field studies did not involve endangered or protected species. Stems were cut into bolts, placed in screened cages, and stored indoors at the University of British Columbia at room temperature. Emerged beetles were collected every three to four days and sexed based on abdominal tergite shape [19].

### Chemicals

Chemicals obtained from Sigma-Aldrich (Mississauga, ON, Canada) were: *N*,*O*-*bis*(trimethylsilyl)trifluoroacetamide (BSTFA, cat. No. 15209), methyl *tert*-butyl ether (MTBE, cat. No. 650560), pentane (cat. No. 34956), (+)-α-pinene (cat. No. P45680, purity 98%, optical purity ee: >88%), and (–)-α-pinene (cat. No. 274399, purity 99%, optical purity ee: >86%), (+)-β-pinene (cat. No. 80607, purity 98%), (–)-β-pinene (cat. No. 112089, purity 99%), (+)-3-carene (cat. No. 441619, purity 99%), (–)-limonene (cat. No. 218367, purity 97%), (+)-limonene (cat. No. 62122, purity ~90%), myrcene (cat. No. M100005, purity 99%), terpinolene (cat. No. 86485, purity >85%), (1*SR*,2*RS*,4*S*)-limonene-1,2-epoxide (cat. No. 218332, purity 99%, optical purity ee: >99%), (1*S*,2*S*,4*R*)-(+)-limonene-1,2-diol (cat. No. W440900, purity >97%), (S)-(–)-perilla alcohol (cat. No. 218391, purity 96%), (4*R*, 6*RS*)-(–)-carveol (cat. No. 192384, purity >97%), and β-nicotinamide adenine dinucleotide 2’-phosphate reduced tetrasodium salt hydrate (NADPH, cat. No. N7505). Chemicals obtained from Helix Biotech (now Cansyn) (Richmond, BC, Canada) were: abietic acid (cat. No. R002, purity 90–95%), dehydroabietic acid (cat. No. R001, purity 99%), neoabietic acid (cat. No. R003, purity 99%), levopimaric acid (cat. No. R005, purity 95%), and isopimaric acid (cat. No. R011, purity 99%). *trans*-Verbenol (~20(+):80(–) optical purity) (lot. No. W06-00141) was obtained from PheroTech (Delta, BC, Canada). (4*R*,8*RS*)-Limonene-8,9-epoxide (cat. No. ZEP0020) was obtained from Endeavour Speciality Chemicals (Daventry, UK). (–)-β-Phellandrene (purity 84%) was obtained by purification from lodgepole pine turpentine by Synergy Semiochemicals (Delta, BC, Canada).

### Identification and heterologous expression of CYP6DJ1, CYP6BW1 and CYP6BW3

*CYP6DJ1* (JQ855677), *CYP6BW1* (JQ855661) and *CYP6BW3* (JQ855663) were identified in the MPB transcriptome [20] as described in [17]. The full open reading frames of the pDNR-LIB EST clones *CYP6DJ1* (DPO0411_I13), *CYP6BW1* (DPO079_G21), *CYP6BW3* (DPO049_N22) were sub-cloned into the pFastBac vector (Invitrogen) and transformed into MAX Efficiency DH10Bac Competent cells (Invitrogen, cat. # 10361–012) to generate recombinant bacmids. Empty pFastBac vector was used to generate a recombinant bacmid for negative controls. Bacmids were used to transfect *Spodoptera frugiperda Sf9* cells (Invitrogen, cat. # 1265–017) for production of baculovirus to a titer of 3–4 x10^7^ IFU mL^-1^. The resulting baculovirus was used to infect 250 mL of Sf9 cell culture (cell density 1.5 x10^6^ cells mL^-1^) at a multiplicity of infection of one. The pelleted seed culture was incubated with the baculovirus culture for 1 h at 27°C and then resuspended in 250 mL of Sf-900 II serum-free media (Invitrogen, cat # 10902–088) with 10% fetal bovine serum. Hemin HCl (Sigma cat #51280) was added to a concentration of 2 μg mL^-1^ 24 h after the infection. Cells were harvested 72 h after infection. Cells were pelleted and washed three times with a 50 mM potassium phosphate buffer solution (KPB), pH7.4. Cells were resuspended in P450 buffer (50 mM of KPB pH 7.4, 20% glycerol, 1 mM EDTA and 0.1 mM DTT), disrupted by sonification, and centrifuged for 1 h at 100,000 x *g* to collect the microsomes. Microsomes were suspended in 5 mL of P450 buffer and aliquots were frozen at -80°C until use. To test for the presence of the P450 proteins, 5 μL of denatured microsomes were analyzed on a 12% SDS-PAGE gel, and P450 activity was checked by carbon monoxide (CO)-difference spectrum analysis [21,22]. MPB CPR (JQ855639) was expressed in *E*. *coli* as previously described [15].

### Enzyme assays

*In vitro* assays with CYP6DJ1, CYP6BW1 and CYP6BW1 were performed individually with each of ten different monoterpenes [(+)-α-pinene, (–)-α-pinene, (+)-β-pinene, (–)-β-pinene, (+)-limonene, (–)-limonene, (+)-3-carene, myrcene, β-phellandrene, and terpinolene] and five different DRAs (abietic acid, dehydroabietic acid, neoabietic acid, levopimaric acid, and isopimaric acid). Assays with microsomes from empty vector expression, as well as assays with P450 microsomes without NADPH, were used as negative controls. Each assay was replicated at least three times. Microsomes from P450 or empty vector expression were combined with CPR microsomes and kept on ice for 1 h before being used in assays. Assays were prepared as follows: 25 μL of P450 microsome (0.5–2.0 μM) and 2 μL of CPR microsome (1U mL^-1^) were added to 2 mL amber glass vials (Agilent, cat# 5182–00716), and KPB (pH 7.4) and NADPH were added for a final concentration of 50 mM KPB and 1 mM NADPH. To start the assays, 3 μL of individual monoterpenes (10 mM in pentane) or DRAs (1 mM in MTBE) was added and the vial was immediately capped. The total assay volume was 300 μL. Assays were incubated for 1 h at 30°C and then extracted three times with pentane (for assays with monoterpenes) or MTBE (for assays with DRAs). Extracts were concentrated under a N_2_ stream to 300 μL. Extracts from assays with DRAs were derivatized by adding 5 μl of BSTFA to 50 μL of assay extract and letting the sample incubate overnight. Assay products were analyzed by gas chromatography coupled mass spectroscopy (GC-MS).

### Beetle treatment with limonene and terpinolene

Female emergent beetles were exposed to vapours of (+)-limonene, (–)-limonene or terpinolene corresponding to 0.05 μL ((+) and (–)-limonene) or 0.1 μL (terpinolene) volume of monoterpene per mL of airspace as described in [4]. A 1.5 cm x 1.5 cm Whatman filter paper was placed into a 20 mL scintillation vial (VWR) and 1 μL of (+) or (–)-limonene or 2 μL of terpinolene was added to the filter paper. For controls, 1 μL of acetone was used instead of monoterpene. Females were placed into the vials with one beetle per vial, and vials were capped. After 24 h, living females were collected, frozen with liquid N_2_ and kept at 80°C until extraction. Each beetle was extracted individually with MTBE and the products analyzed by GC-MS. Each replicate consisted of a single extracted female beetle and four replicates were performed per monoterpene treatment. Frozen beetles were crushed individually in a 2.0 ml Safe-Lock tube (Eppendorf) over dry ice, using a cold glass stir rod, in 0.5 ml of MTBE containing 1 ng μL^-1^ of tridecane added as an internal standard. The crushed beetle was removed from dry ice, allowed to thaw for a few minutes and centrifuged for 20s at 2000 *x g*. The crushed beetle was then frozen again on dry ice before the MTBE was transferred to an amber 2 mL glass vial (Agilent). This process was repeated with another 0.5 ml of MTBE. Extracts of beetles were treated to remove excess fatty acids from the sample as follows: 400 μL of 1 mM ammonium carbonate (pH 8) was added to the combined MTBE extract and vortexed. The sample was centrifuged for 10 min at 3000 x *g* and the MTBE layer removed for analysis by GC-MS.

### GC-MS analysis

GC-MS analyses of monoterpenoids were performed on an Agilent 7890A system GC and a Agilent GC Sampler 80. Monoterpenoids were analyzed by injecting 1 μL of sample onto a DB-WAX column (Agilent J&W, polyethylene glycol, 30 m, 250 mm i.d., 0.25 μm film). Oven temperature for analysis of products from enzyme assays with monoterpenes was 40°C for 2 min, 8°C min^-1^ to 100°C, 20°C min^-1^ to 230°C and then held for 5 min. Oven temperature analysis of beetle extracts treated with monoterpenes was 40°C for 2 min, 8°C min^-1^ to 100°C, 20°C min^-1^ to 250°C and then held for 10 min. GC-MS analyses of diterpenoids were performed on an a 7000A GC-MS triple quad M5975C inert XL MSD with triple axis detector at 70 eV. The 7000A GC-MS triple quad was operated in Full Scan Mode similar to analysis using a single quad. Diterpenoids were analyzed by injecting 1 μL of derivatized sample onto an HP-5 column (Agilent J&W, 5% phenyl methyl siloxane, 27.4 m length, 250 μm i.d., 0.25-μm film thickness). Oven temperature was 40°C for 1 min, 20°C min^-1^ to 300°C and then held for 8 min.

## Results

### CYP6DJ1 uses the monoterpenes (+)-(4R)-limonene, (–)-(4S)-limonene and terpinolene as substrates

**Fig 1:**
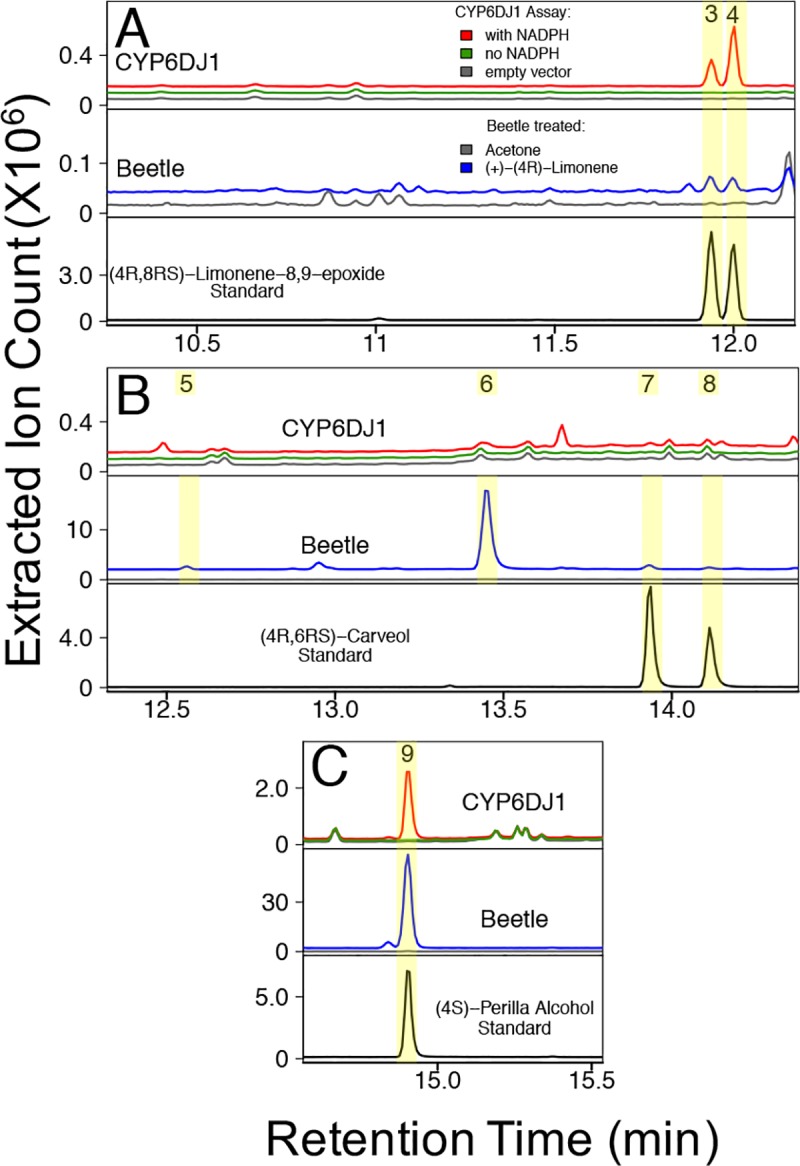
Products of CYP6DJ1 assays with (+)-(4*R*)-limonene and products detected in female beetles after treatment with (+)-(4*R*)-limonene. Panels A-C show different regions of the same chromatograms of CYP6DJ1 assays and extracts of beetles treated with (+)-(4*R*)-limonene. (A) (4*R*,8*R*)-Limonene-8,9-epoxide (peak 3) and (4*R*,8*S*)-limonene-8,9-epoxide (peak 4) were produced by CYP6DJ1 in *in vitro* assays with (+)-(4*R*)-limonene as substrate and were detected in beetles treated with (+)-(4*R*)-limonene. (B) Unidentified product (peak 5), (+)-*trans*-(3*R*,4*S*)-isopiperitenol (peak 6), (+)-*cis*-(4*S*,6*S*)-carveol (peak 7) and (+)-*trans*-(4*S*,6*R*)-carveol (peak 8) were present in beetles treated with (+)-(4*R*)-limonene. (C) (4*R*)-Perilla alcohol (peak 9) was produced in CYP6DJ1 assays with (+)-(4*R*)-limonene and was detected in beetles treated with (+)-(4*R*)-limonene. Chromatograms are shown with the total of the extracted ions 91, 94, 108, 109, 119, 121, 137, 152 m/z. Retention indices and mass spectra of peaks 1–10 are shown in S2 Table and S3 Fig.

**Fig 2:**
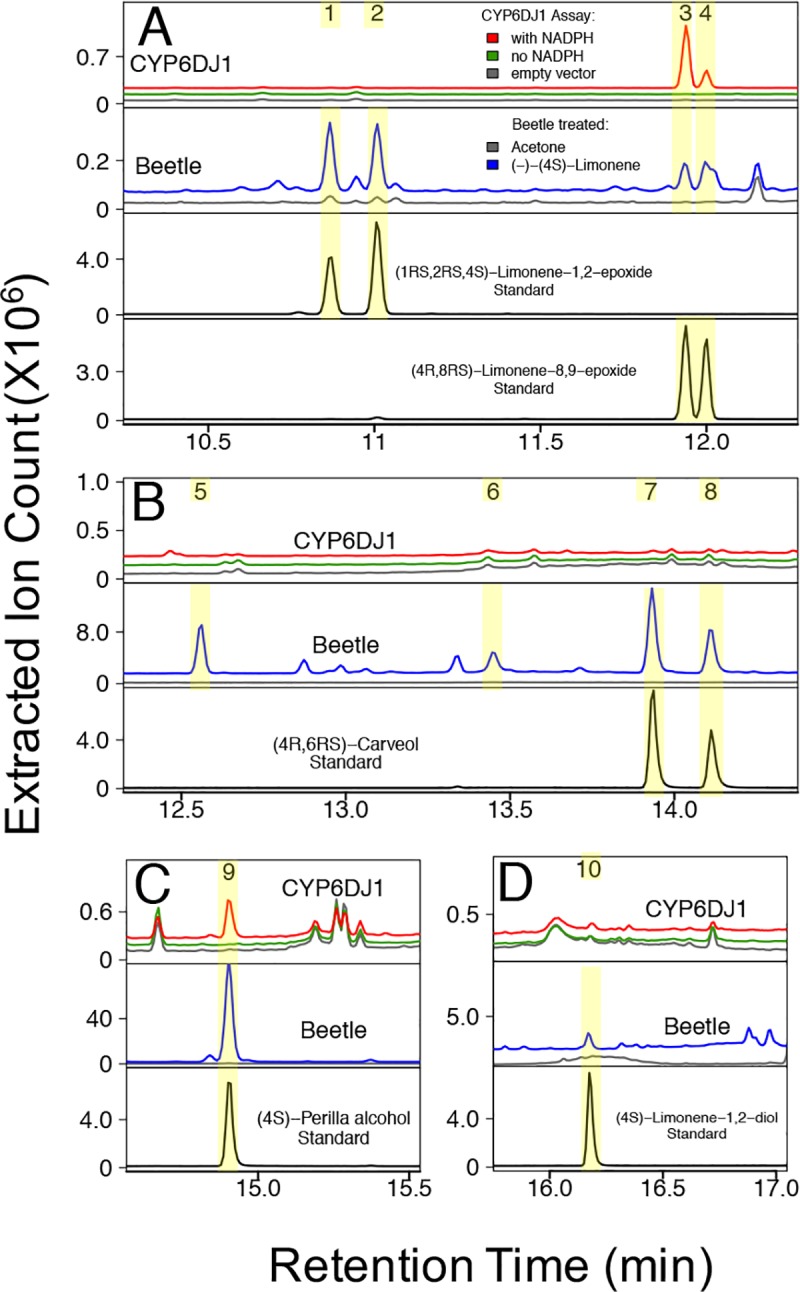
Products of CYP6DJ1 assays with (–)-(4*S*)-limonene and products detected in female beetles after treatment with (–)-(4*S*)-limonene. Panels A-D show different regions of the same chromatograms of CYP6DJ1 assays and extracts of beetles treated with (–)-(4*S*)-limonene. (A) (1*S*,2*R*,4*S*)-Limonene-1,2-epoxide (peak 1) and (1*R*,2*S*,4*S*)-limonene-1,2-epoxide (peak 2) were detected in beetles treated with (–)-(4*S*)-limonene. (4*S*,8*S*)-Limonene-8,9-epoxide (peak 3) and (4*S*,8*R*)-limonene-8,9-epoxide (peak 4) were produced by CYP6DJ1 in *in vitro* assays with (–)-(4*S*)-limonene and were detected in beetles treated with (–)-(4*S*)-limonene. (B) An unidentified product (peak 5), (–)-*trans*-(3*S*,4*R*)-isopiperitenol (peak 6), (–)-*cis*-(4*R*,6*R*)-carveol (peak 7) and (–)-*trans*-(4*R*,6*S*)-carveol (peak 8) were detected in beetles treated with (–)-(4*S*)-limonene. (C) (4*S*)-Perilla alcohol (peak 9) was produced in CYP6DJ1 assays with (–)-(4*S*)-limonene and was detected in beetles treated with (–)-(4*S*)-limonene. (D) (4*S*)-Limonene-1,2-diol was detected in beetles treated with (–)-(4*S*)-limonene. Chromatograms are shown with the total of the extracted ions 91, 94, 108, 109, 119, 121, 137, 152 m/z. Retention indices and mass spectra of peaks 1–10 are shown in S2 Table and S4 and S5 Figs.

**Fig 3:**
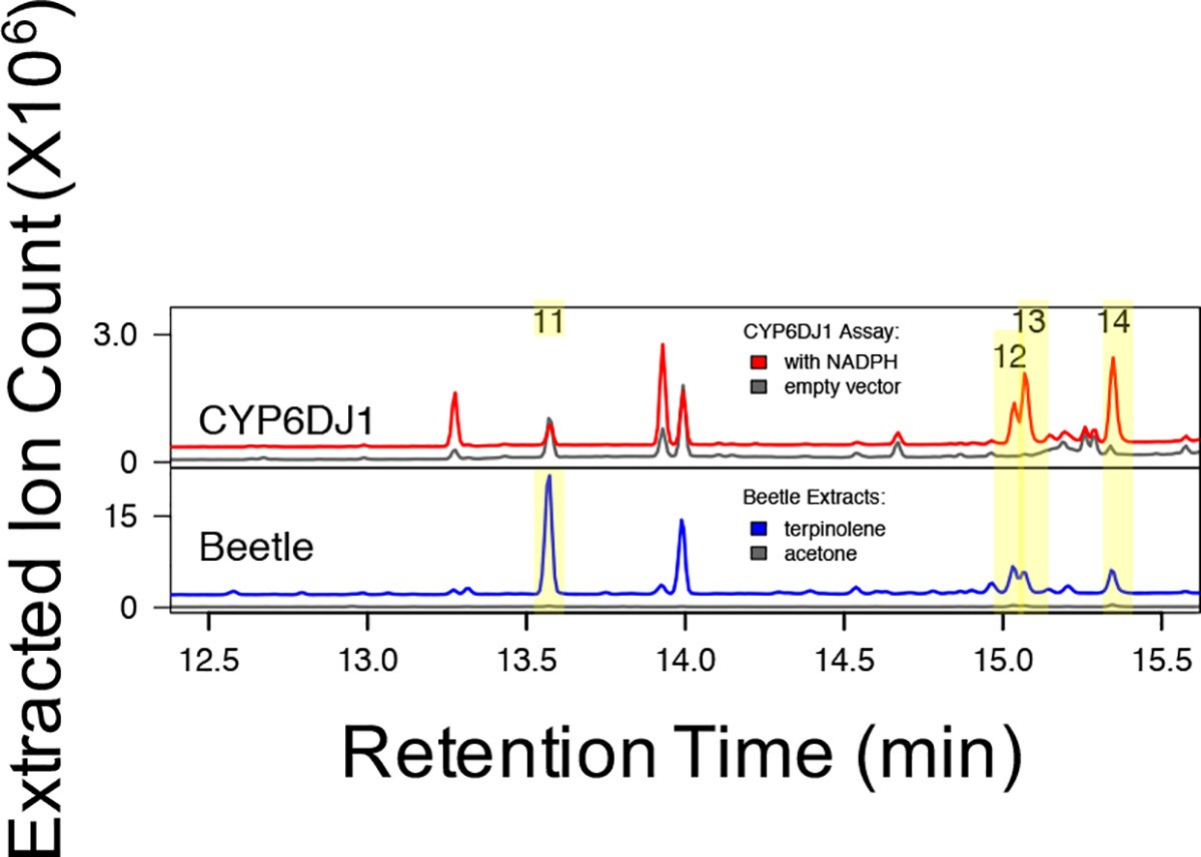
Products of CYP6DJ1 assays with terpinolene and products detected in female beetles after treatment with terpinolene. Four unidentified terpinolene-derived metabolites are present in extracts of terpinolene treated beetles (peaks 11–14), three of which, (peaks 12–14) are found in CYP6DJ1 assays with terpinolene. Chromatograms are shown with the total of the extracted ions 91, 94, 108, 109, 119, 121, 137, 152 m/z. Retention indices and mass spectra of peaks 1–10 are shown in S2 Table and S6 Fig.

CYP6DJ1 was expressed in Sf9 insect cells, isolated as microsomal membrane-bound proteins (**S1 Fig**), and identified as a functional P450 based on its CO-spectrum (**S2 Fig**). CYP6DJ1 was reconstituted with MPB CPR and tested in *in vitro* assays with ten different monoterpenes and six different DRAs (**S1 Table**). The substrates that were tested represent typical monoterpenes and DRAs found in the phloem of the MPB host lodgepole pine [23–25]. CYP6DJ1 was active with three of the ten monoterpenes, specifically the two enantiomers of limonene as well as terpinolene (**Figs 1–3, S1 Table**). CYP6DJ1 was not active with the other seven monoterpenes tested nor with any of the DRAs tested (**S1 Table**).

### Products formed by CYP6DJ1 with (+)-(4R)-limonene, (–)-(4S)-limonene or terpinolene as substrates

**Fig 4:**
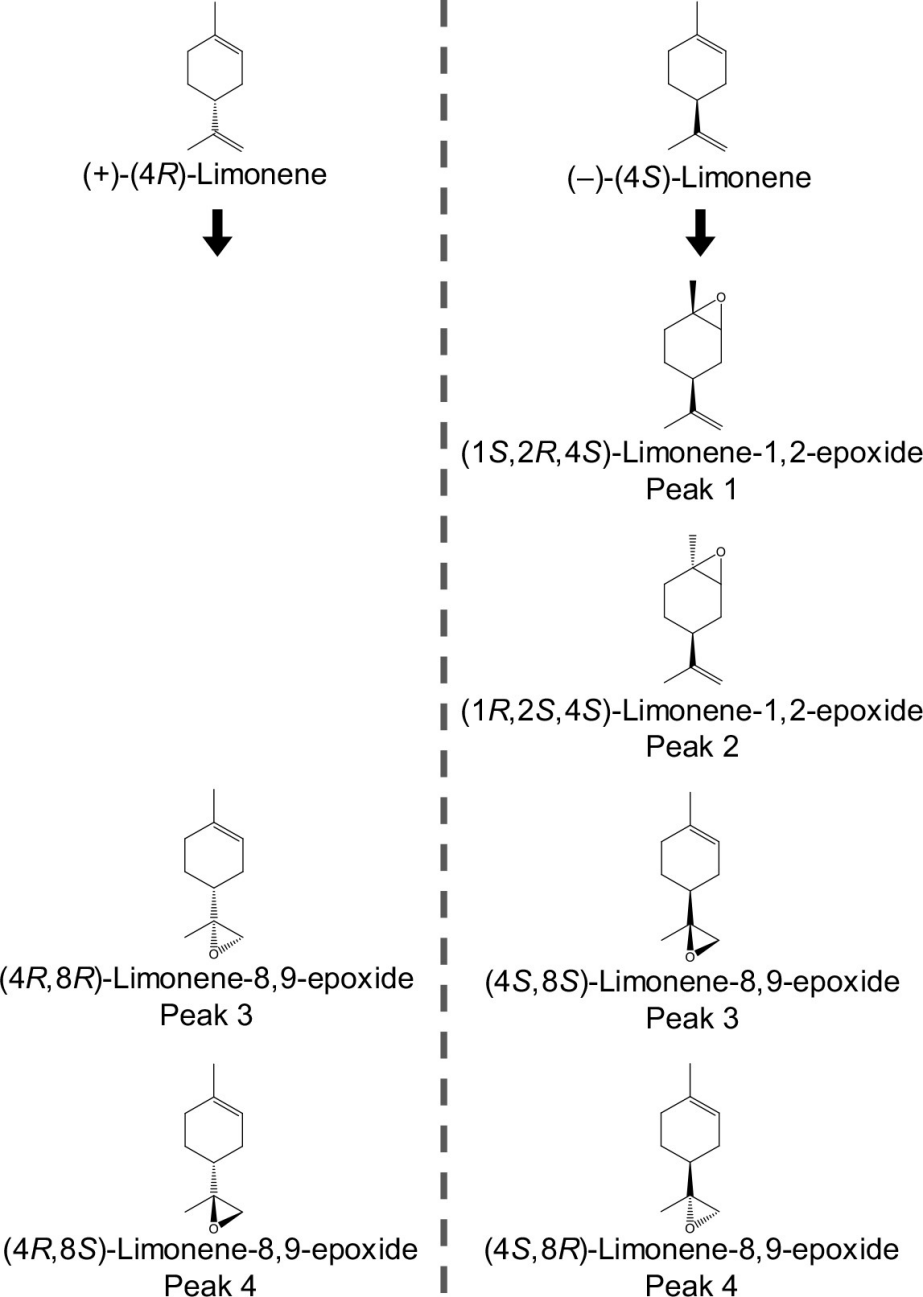
Chemical structure of the epoxide products (peak 1–4) of (+)-(4*R*)-limonene and (–)-(4*S*)-limonene identified in CYP6DJ1 assays and extracts of beetles treated with these monoterpenes. Enantiomers are shown directly across the dotted line from each other. (4*R*,8*R*)-Limonene-8,9-epoxide and (4*S*,8*S*)-limonene-8,9-epoxide are enantiomers and both eluted as peak 3 in an achiral column. (4*R*,8*S*)-Limonene-8,9-epoxide and (4*S*,8*R*)-limonene-8,9-epoxide are enantiomers and both eluted as peak 4.

**Fig 5:**
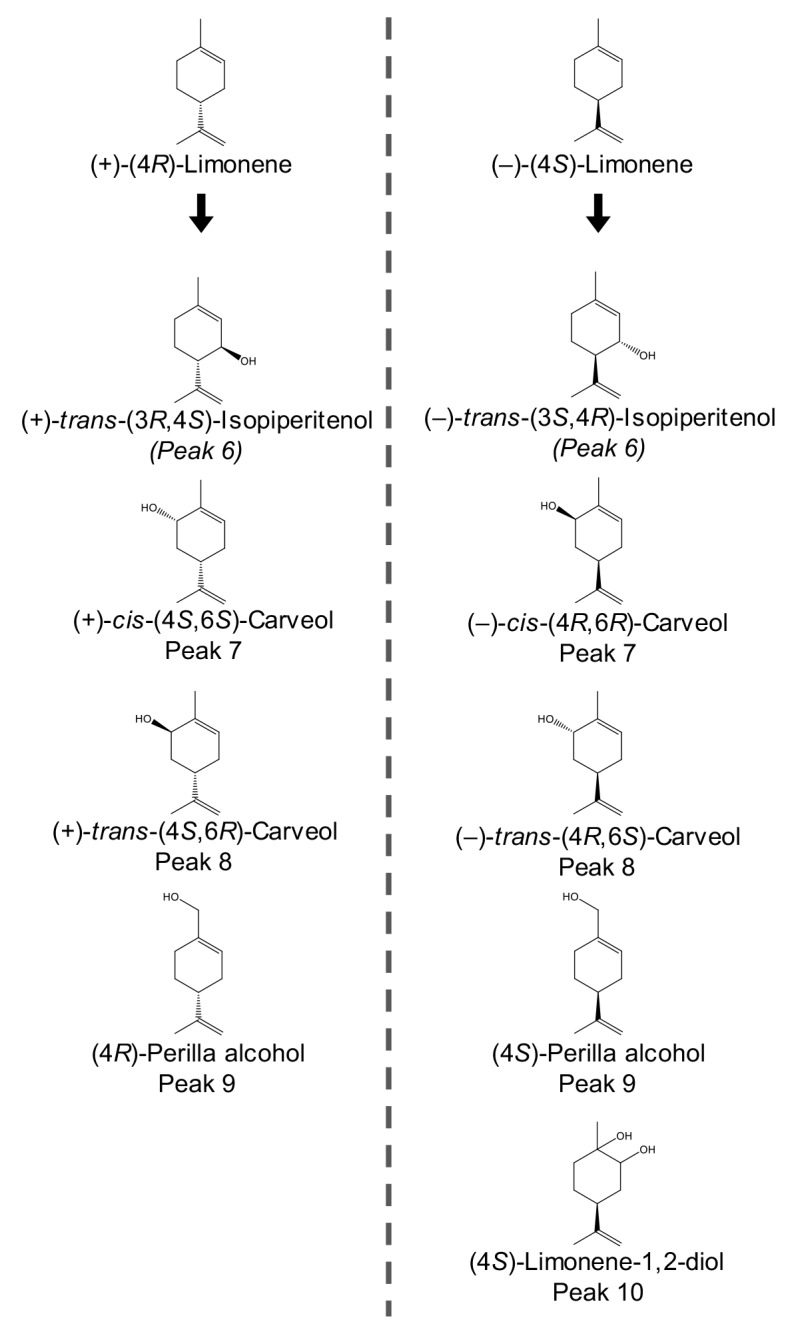
Chemical structure of the alcohol products (peak 6–10) products of (+)-(4*R*)-limonene and (–)-(4*S*)-limonene identified in CYP6DJ1 assays and extracts of beetles treated with these monoterpenes. Enantiomers are shown directly across the dotted line from each other. (+)-*trans*-(3*R*,4*S*)-Isopiperitenol and (–)-*trans*-(3*S*,4*R*)-isopiperitenol are enantiomers and the proposed products of peak 6. (+)-*cis*-(4*S*,6*S*)-Carveol and (–)-*cis*-(4*R*,6*R*)-carveol are enantiomers and both eluted as peak 7. (+)-*trans*-(4*S*,6*R*)-Carveol and (–)-*trans*-(4*R*,6*S*)-carveol are enantiomers and both eluted as peak 8. (4*R*)-Perilla alcohol and (4*S*)-perilla alcohol are enantiomers and both eluted as peak 9.

Products of CYP6DJ1 assays with limonene or terpinolene were identified by comparison of retention times (**Figs 1–3, S2 Table**) and mass-spectra (**S3–S6 Figs**) with those of authentic standards. The products of CYP6DJ1 assays with (+)-(4*R*)-limonene were identified as (4*R*,8*R*)-limonene-8,9-epoxide (peak 3), (4*R*,8*S*)-limonene-8,9-epoxide (peak 4) and (4*R*)-perilla alcohol (peak 9) (**Fig 1, Figs 4 and 5, S3 Fig**). Products of CYP6DJ1 assays with (–)-(4*S*)-limonene were (4*S*,8*S*)-limonene-8,9-epoxide (peak 3), (4*S*,8*R*)-limonene-8,9-epoxide (peak 4) and (4*S*)-perilla alcohol (peak 9) (**Fig 2, Figs 4 and 5, S4 and S5 Figs**). The limonene-8,9-epoxides (peak 3 and 4), and perilla alcohols (peak 9) were not detected in control assays with protein expressed from empty vector, or in control assays that did not contain NADPH (**Fig 2**). Diastereomers present in the (–)-(1*RS*,2*RS*,4*S)-*limonene-1,2-epoxides, (4*R*,8*RS*)-limonene-8,9-epoxide and (–)-(4*R*,6*RS*)-carveols standards were identified based on published retention indices [26,27]. Some products of (+)-(4*R*)-limonene and (–)-(4*S*)-limonene identified in CYP6DJ1 assays and extracts of MPB were enantiomers and had the same retention index on the DB-WAX column (**Figs 4 and 5, S2 Table**). Assays with CYP6DJ1 and terpinolene produced three unidentified peaks (peak 12–14) (**Fig 3 and S6 Fig**). Peaks 12, 13 and 14 were not detected in assays with the empty vector control, or in assays that did not contain NADPH.

### Oxidized monoterpenes produced in MPB exposed to (+)-(4R)-limonene, (–)-(4S)-limonene or terpinolene

Female beetles were exposed to the monoterpenes (+)-(4*R*)-limonene, (–)-(4*S*)-limonene and terpinolene, and the products extracted from these beetles were compared to those found in the CYP6DJ1 *in vitro* assays. Extracts of beetles exposed to (+)-(4*R*)-limonene contained (4*R*,8*R*)-limonene-8,9-epoxide (peak 3), (4*R*,8*S*)-limonene-8,9-epoxide (peak 4), an unidentified product (peak 5), (+)-*trans*-(3*R*,4*S*)-isopiperitenol (peak 6), (+)-*cis*-(4*S*,6*S*)-carveol (peak 7), (+)-*trans*-(4*S*,6*R*)-carveol (peak 8) and (4*R*)-perilla alcohol (peak 9) (**Fig 1, Figs 4 and 5, S3 Fig**). These metabolites were not present in females that had been exposed to acetone as a control (**Fig 1**). Extracts of beetles exposed to (–)-(4*S*)-limonene contained (1*S*,2*R*,4*S*)-limonene-1,2-epoxide (peak 1), (1*R*,2*S*,4*S*)-limonene-1,2-epoxide (peak 2), (4*S*,8*S*)-limonene-8,9-epoxide (peak 3), (4*S*,8*R*)-limonene-8,9-epoxide (peak 4), unidentified product (peak 5), (–)-*trans*-(3*S*,4*R*)-isopiperitenol (peak 6), (–)-*cis*-(4*R*,6*R*)-carveol (peak 7), (–)-*trans*-(4*R*,6*S*)-carveol (peak 8), (4*S*)-perilla alcohol (peak 9), and (4*S*)-limonene-1,2-diol (**Fig 2, Figs 4 and 5, S4 and S5 Figs**). These metabolites were not present in control females exposed to acetone (**S2 Fig**). Lacking an authentic standard for peak 6, we identified peak 6 as (+)-*trans*-(3*R*,4*S*)-isopiperitenol and (–)-*trans*-(3*S*,4*R*)-isopiperitenol based on matching retention index and mass spectra to that reported in [27,28]. Extracts of female beetles that had been exposed to terpinolene contained four unidentified products, peaks 11–14 (**Fig 3 and S6 Fig**). These metabolites were not present in control females exposed to acetone (**Fig 3**).

### Comparison of *in vitro* products of CYP6DJ1 and metabolites detected in MPB exposed *in vivo* to (+)-(4R)-limonene, (–)-(4S)-limonene or terpinolene

**Fig 6:**
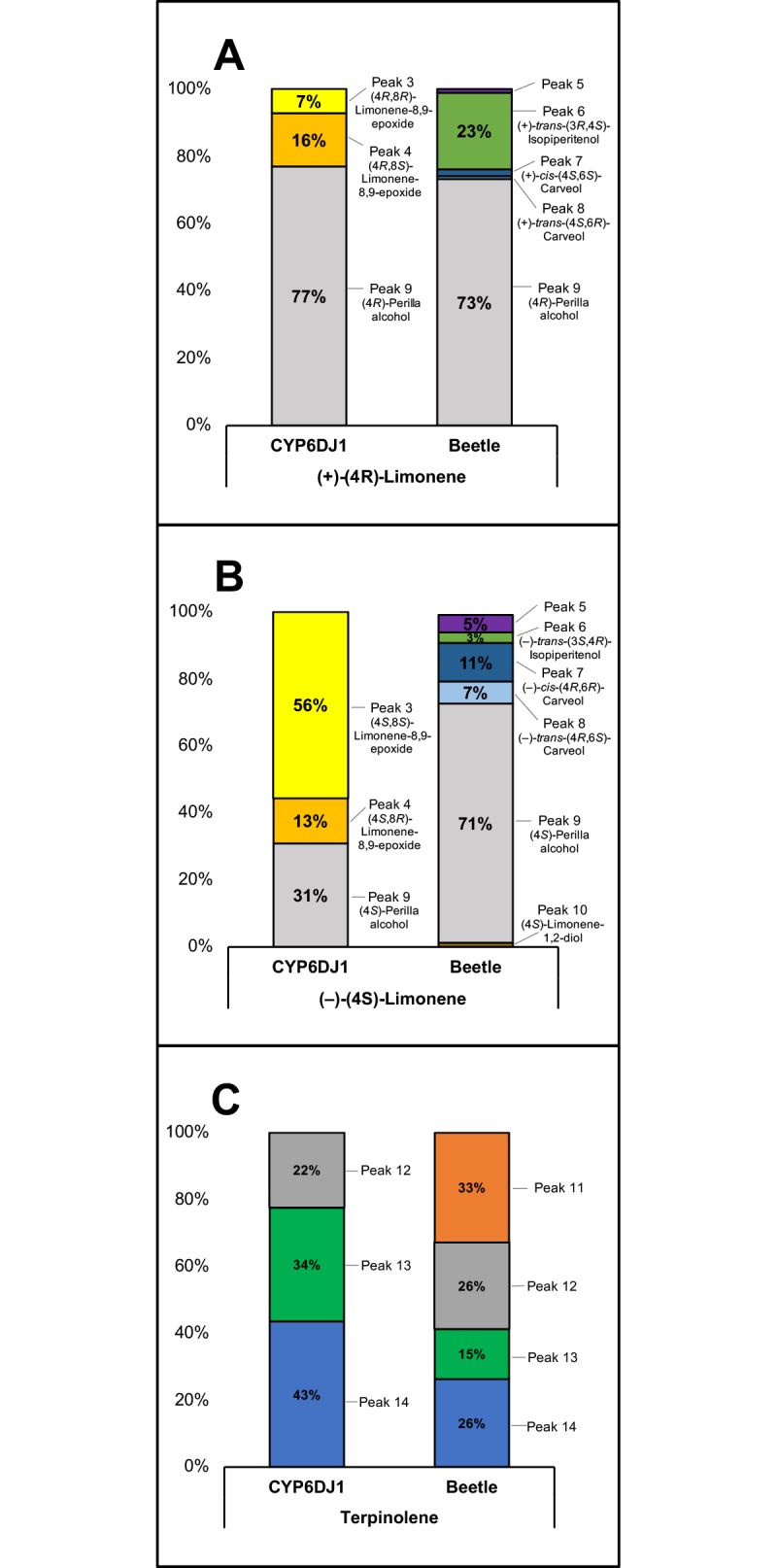
Product profiles of recombinant CYP6DJ1 with (+)-(4*R*)-limonene, (–)-(4*S*)-limonene or terpinolene as substrates and metabolites detected in extracts of beetles treated with (+)-(4*R*)-limonene, (–)-(4*S*)-limonene or terpinolene. The relative abundance (percentage) across the profiles was calculated by peak area of the extracted ion chromatogram. Retention indices and mass spectra are shown in S2 Table and S3–S5 Figs. CYP6DJ1 profile N = 3, Beetle profile N = 3.

The activity of CYP6DJ1 with (+)-(4*R*)-limonene, (–)-(4S)-limonene, and terpinolene resulted in multiple products for each substrate. We measured the relative amounts of each product formed by CYP6DJ1 in *in vitro* assays and compared them with those detected in extracts of beetles exposed to (+)-(4*R*)-limonene, (–)-(4S)-limonene, and terpinolene (**Fig 6**).

The product profile of CYP6DJ1 with (+)-(4*R*)-limonene as substrate consisted of the major product (77%) (4*R*)-perilla alcohol (peak 9), followed by 16% (4*R*,8*S*)-limonene-8,9-epoxide (peak 4), and 7% (4*R*,8*R*)-limonene-8,9-epoxide (peak 3) (**Fig 6A**). The product profile of (+)-(4*R*)-limonene-treated beetles was also dominated by 73% (4*R*)-perilla alcohol (peak 9), with additional metabolites in order of decreasing abundance 23% (+)-*trans*-(3*R*,4*S*)-isopiperitenol (peak 6), 2% (+)-*cis*-(4*S*,6*S*)-carveol (peak 7), 1% each of (+)-*trans*-(4*S*,6*R*)-carveol (peak 8) and an unknown compound (peak 5), as well as <1% each of (4*R*,8*R*)-limonene-8,9-epoxide (peak 3) and (4*R*,8*S*)-limonene-8,9-epoxide (peak 4) (**Fig 6A**).

The product profile of CYP6DJ1 with (–)-(4S)-limonene consisted of the major product (56%) (4*S*,8*S*)-limonene-8,9-epoxide (peak 3), together with 31% (4*S*)-perilla alcohol (peak 9) and 13% (1*R*,2*S*,4*S*)-limonene-1,2-epoxide (peak 4) (**Fig 6B**). The metabolite profile of (–)-(4*S*)-limonene beetle was substantially different, with the major product (71%) (4*S*)-perilla alcohol (peak 9), followed by 11% (–)-*cis*-(4*R*,6*R*)-carveol (peak 7), 7% (–)-*trans*-(4*R*,6*S*)-carveol (peak 8), 5% peak 5, 3% (–)-*trans*-(3*S*,4*R*)-isopiperitenol (peak 6), and 3% (4*S*)-limonene-1,2-diol (peak 10) (**Fig 6B**). In addition, (1*S*,2*R*,4S)-limonene-1,2-epoxide (peak 1), (1*R*,2*S*,4*S*)-limonene-1,2-epoxide (peak 2), (4*S*,8*S*)-limonene-8,9-epoxide (peak 3), and (4*S*,8*R*)-limonene-8,9-epoxide (peak 4) together accounted for less than 2% of the metabolite profile in beetle extracts (**Fig 6**).

The product profile of CYP6DJ1 with terpinolene consisted of the major product (43%) peak 14, followed by 34% peak 13 and 22% peak 12 (**Fig 6C**). The metabolite profile of terpinolene beetle contained the major product (33%) peak 11, followed by 26% peak 12, 26% peak 14, and 15% peak 13 (**Fig 6C**).

### CYP6BW1 and CYP6BW3 are active with diterpene resin acids as substrates

CYP6BW1 and CYP6BW3 were produced in Sf9 insect cells, isolated as microsomal membrane-bound proteins (**S1 Fig**), and identified as functional P450s based on CO-spectra (**S2 Fig**). CYP6BW1 and CYP6BW3 were reconstituted with MPB CPR and tested in *in vitro* enzyme assays with ten different monoterpenes and six different DRAs (**S1 Table**). Both CYP6BW1 and CYP6BW3 oxidized all six diterpene resin acids but were not active with any of the monoterpenes tested.

### Products formed by CYP6BW1 and CYP6BW3 with diterpene resin acids

**Fig 7:**
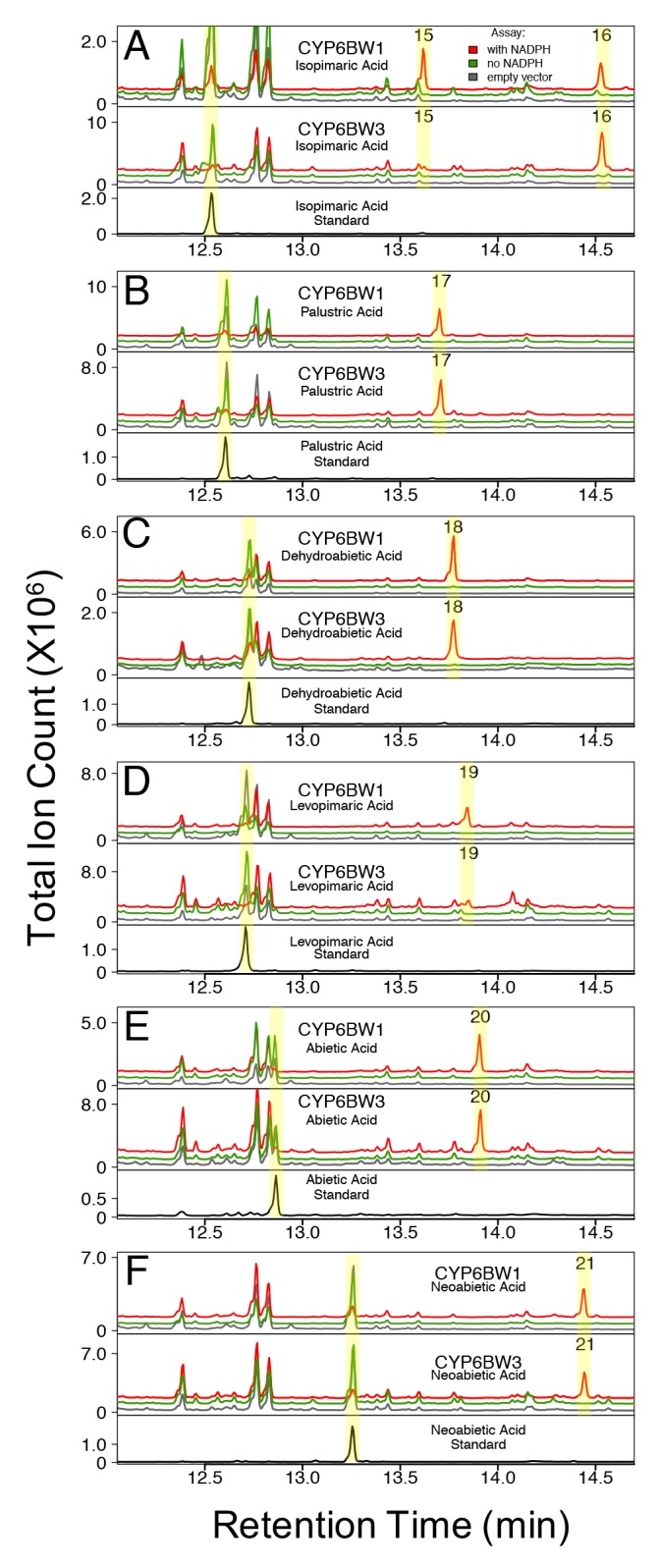
Products of recombinant CYP6BW1 and CYP6BW3 with diterpene resin acids. CYP6BW1 and CYP6BW3 assays with (A) isopimaric acid (B) palustric acid, (C) dehydroabietic acid, (D) levopimaric acid, (E) abietic acid, (F) neoabietic acid, as a substrate. Retention indices and mass spectra of peaks 15–21 are shown in S3 Table and S7 and S8 Figs.

Overall, CYP6BW1 and CYP6BW3 gave similar product profiles (peaks 15–21) with each of the six different DRAs (**Fig 7**). Since authentic standards were not available for compounds corresponding to peaks 15–21, we used mass spectra to deduce product identities. BSTFA was used to derivatize products of *in vitro* assays prior to GC-MS analysis; therefore, free hydroxyl groups were replaced by trimethylsilyl ether, adding an additional 72 m/z to the mass for each derivatized hydroxy group, including that of the carboxylic acid group.

In assays of CYP6BW1 and CYP6BW3 with isopimaric acid, we found two product peaks (peak 15 and 16), with peak 15 in the CYP6BW3 assay as a very minor component (**Fig 7A**). Peak 15 had a mass spectrum with a molecular ion of 390 m/z (**S7 Fig**), consistent with a singly epoxidized isopimaric acid. Peak 16 had a mass spectrum with a fragment ion of 537 m/z (**S7 Fig**), consistent with an [M-15]^+^ of a TMS-derivatized vicinal diol due to the hydrolysis of the epoxidized isopimaric acid.

CYP6BW1 and CYP6BW3 produced single products, corresponding to peaks 17–21, with palustric acid, dehydroabietic acid, levopimaric acid, abietic acid, and neoabietic acid, respectively (**Fig 7B–7F**). The mass spectrum of peak 18 had a molecular ion of m/z 460 while the mass spectra of peak 17, 19, 20 and 21 had a molecular ion of m/z 462 (**S8 Fig**). These molecular ions are consistent with a singly hydroxylated substrate.

Hydroxylated and epoxidized products of the DRAs were not detected in assays with the empty vector control, or with CYP6BW1 and CYP6BW3 assays that did not contain NADPH, an important co-factor for P450 activity. CYP6BW1 and CYP6BW3 were not active with the ten monoterpenes tested (**S1 Table**). We obtained a sample of a 15-hydroxyabietic acid compound that had been previously identified [29] but although it had a similar mass spectrum to our hydroxylated abietic acid compound, this compound did not co-elute with any of the products of CYP6BW1 and CYP6BW3 assays.

## Discussion

CYP6DJ1 oxidized (+)-limonene, (–)-limonene, and terpinolene. When either enantiomer of limonene was presented as a substrate, CYP6DJ1 produced limonene-8,9-epoxide and perilla alcohol. Perilla alcohol was also the major product in beetles, while limonene-8,9-epoxide was present only in trace amounts. These results suggested that CYPDJ1 may contribute to the metabolism of limonene in the beetle. However, the presence of other limonene metabolites in beetles, which were not products with CYP6DJ1, suggests that additional enzymes may also contribute to the metabolism of limonene *in vivo*. Limonene has particularly high toxicity to MPB compared to other monoterpenes, with (–)-limonene being significantly more toxic than (+)-limonene [4,30]. In addition, the presence of (+)-limonene in host volatiles has been implicated in the disruption of host selection by female MPB [31]. However, despite having a potentially higher defensive potential against MPB attack, limonene accounts for only a minor percentage of the monoterpene profile of pine host species for MPB [23,25,32].

It is assumed is that the P450 oxidation products of pine monoterpenes may be less toxic than the monoterpenes themselves. However, without future testing, it cannot be excluded that the oxidized monoterpene products remain toxic or could potentially be even more toxic than the monoterpene olefins of the pine oleoresin. Evidence of herbivore metabolism producing potentially more toxic compounds from plant defense metabolites exists, as for example with pyrrolizidine alkaloids [33,34]. In the MPB-associated fungus, *Grosmannia clavigera*, the limonene oxidation products, carveol, perilla alcohol and limonene epoxide were shown to be more toxic than limonene itself [6]. As previously shown, the limonene oxidation products are further modified by the MPB, specifically the limonene derived monoterpenol carveol and perilla alcohol are further metabolized to monoterpenyl esters [5].

Previous transcriptome and proteome analyses indicated that the *CYP6DJ1* transcript and CYP6DJ1 protein are upregulated in female MPB compared to males during the host colonization process and feeding [16,17,35] or treatment with juvenile hormone III [36]. The upregulation of CYP6DJ1 in colonizing females might have suggested that it could be involved in the formation of *trans*-verbenol, an aggregation pheromone of female MPB derived from the oxidation of α-pinene. However, CYP6DJ1 did not show activity with α-pinene. We recently showed that *trans*-verbenol released from colonizing females is derived from verbenyl esters accumulated by females earlier in the life cycle [5]. The production of the pheromone *trans*-verbenol via α-pinene hydroxylation may not be occurring in colonizing adult females, as previously thought. Therefore, a P450 involved in *trans*-verbenol biosynthesis specifically for pheromone production may not be expected to be upregulated in these females. A role of CYP6DJ1 in detoxifying limonene and terpinolene may be more important to colonizing females than males, since females are the pioneer sex and the first to contend with host defenses.

We showed that both CYP6BW1 and CYP6BW3 oxidize the same set of major DRAs of the pine oleoresin and produce mostly the same hydroxylated and epoxidized products. Future research will need to identify these products and their presence in MPB exposed to DRAs to assess their biological role in MPB. Since CYP6BW1 and CYP6BW3 share 96% amino acid identity, it is not surprising that these two enzymes were active with the same DRAs. However, their biological roles in the beetle are likely different, as CYP6BW1 transcript is highly expressed in the midgut whereas CYP6BW3 transcript is highly expressed in the antennae [17,20,36]. CYP6BW1 may be metabolizing DRAs as part of general detoxification in the midgut, while CYP6BW3 may have a more specialized role in the protection of the olfactory system in the antennae. Putative orthologues of MPB CYP6BW1 and CYP6BW3 have been identified in other *Dendroctonus* species, sharing over 90% amino acid identity to CYP6BW1 and CYP6BW3. *CYP6BW5v1* from *D*. *rhizophagus* and *CYP6BW5v3* from *D*. *valens* appear to be single copy genes that are expressed in both the antennae and midgut [37,38]. *CYP6BW1* and *CYP6BW3* may have evolved by gene duplication from a single copy gene that was expressed in both the antennae and midgut and may have subsequently diverged with different patterns of expression indicative of sub-functionalization. Previous transcriptome analyses also identified *CYP6BW3* transcripts as upregulated in colonizing males, compared to colonizing females, which may have suggested a role in the biosynthesis of the male produced pheromone frontalin [16,17]. Our results do not support such a function.

Overall, the activity of CYP6DJ1 with both enantiomers of limonene as well as terpinolene as substrates, and the activity of CYP6BW1 and CYP6BW3 with six different DRAs as substrates, provides the MPB with substantial P450 biochemical capacities to cope with pine chemical defenses. Together with other terpene oxidizing MPB P450s, namely CYP345E2, CYP6DE1 and CYP6DE3 [13,15,16], these enzymes may contribute to the survival of MPB in the terpenoid oleoresin saturated environment of the host phloem [13,15,16][13,15,16][13,15,16][13,15,16][13,15,16].

### GenBank accessions

GenBank accession numbers for the MPB P450 described in this paper are *CYP6DJ1* (JQ855677, DPO0411_I13), *CYP6BW1* (JQ855661, DPO079_G21) and *CYP6BW3* (JQ855663, DPO049_N22).

## Supporting information

S1 Fig Denatured CYP6DJ1, CYP6BW1, CYP6BW3 and empty vector control microsomes on a 12% SDS-Page gel. Lane 1: Precision Plus Protein ladder (Bio-rad). Lane 2: empty vector microsomes. Lane 3: CYP6BW1 microsomes, protein band is visible between 75 kDa and 50 kDa. Lane 4: CYP6BW3 microsomes, protein band is visible between 75 kDa and 50 kDa. Lane 5: Precision Plus Protein ladder (Bio-rad). Lane 6: empty vector microsomes. Lane 7: CYP6DJ1 microsomes, protein band is visible between 75 kDa and 50 kDa.
(PDF)

S2 Fig CO Spectra of CYP6DJ1, CYP6BW1, CYP6BW3 and empty vector control microsomes. (PDF)

S3 Fig Mass spectra of peaks 3–9 from the gas chromatograms of extracts of CYP6DJ1 or female beetles treated with (+)-(4*R*)-limonene along with the standards. Gas chromatograms with peak numbers can be found in Fig 1.
(PDF)

S4 Fig Mass spectra of peaks 1–8 from the gas chromatograms of extracts of CYP6DJ1 or female beetles treated with (–)-(4*S*)-limonene along with the standards. Gas chromatograms with peak numbers can be found in Fig 2.
(PDF)

S5 Fig Mass spectra of peaks 9 and 10 from the gas chromatograms of extracts of CYP6DJ1 or female beetles treated with with (–)-(4*S*)-limonene along with the standards. Gas chromatograms with peak numbers can be found in Fig 2.
(PDF)

S6 Fig Mass spectra of peaks 11–14 from the gas chromatograms of extracts of CYP6DJ1 or female beetles treated with terpinolene. Gas chromatograms with peak numbers can be found in Fig 3.
(PDF)

S7 Fig Mass spectra of peaks 15–18 from the gas chromatograms of extracts of CYP6BW1 or CYP6BW3 or female beetles treated with isopimaric, palustric, or dehydroabietic acid. Gas chromatograms with peak numbers can be found in Fig 7A–7C.
(PDF)

S8 Fig Mass spectra of peaks 19–21 from the gas chromatograms of extracts of CYP6BW1 or CYP6BW3 or female beetles treated with levopimaric, abietic or neoabietic acid. Gas chromatograms with peak numbers can be found in Fig 7D–7F.
(PDF)

S1 Table Activity assay of CYP6DJ1, CYP6BW1 and CYP6BW3 with ten selected monoterpenes and six diterpene resin acid substrates. Red crosses indicate that no product was detected in GC chromatograms of the assay, green checkmarks indicate that one or more products were detected in the assay.
(PDF)

S2 Table The retention index of all limonene and terpinolene products of CYP6DJ1 and from extracts of MPB after treatment. All samples were injected onto a DB-Wax column. See Figs 1–5 and S3–S6 Figs. for the gas chromatograms, structures and mass spectra of these peaks.
(PDF)

S3 Table The retention index of diterpene resin acid products of CYP6BW1 and CYP6BW3 and from extracts of MPB after treatment. All samples were injected onto a HP-5 column. See Fig 7 and S7 and S8 Figs for the gas chromatograms and mass spectra of these peaks.
(PDF)
