# Supplementary figures and images for: Functions of mountain pine beetle cytochromes P450 CYP6DJ1, CYP6BW1 and CYP6BW3 in the oxidation of pine monoterpenes and diterpene resin acids
Source: PLoS One. 2019 May 9;14(5):e0216753. doi: 10.1371/journal.pone.0216753 (PMC6508646; doi:10.1371/journal.pone.0216753)

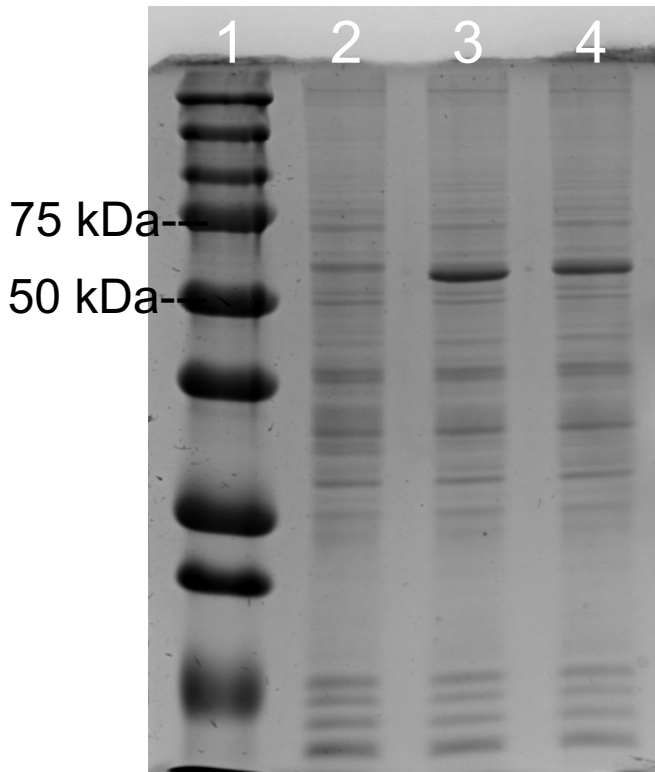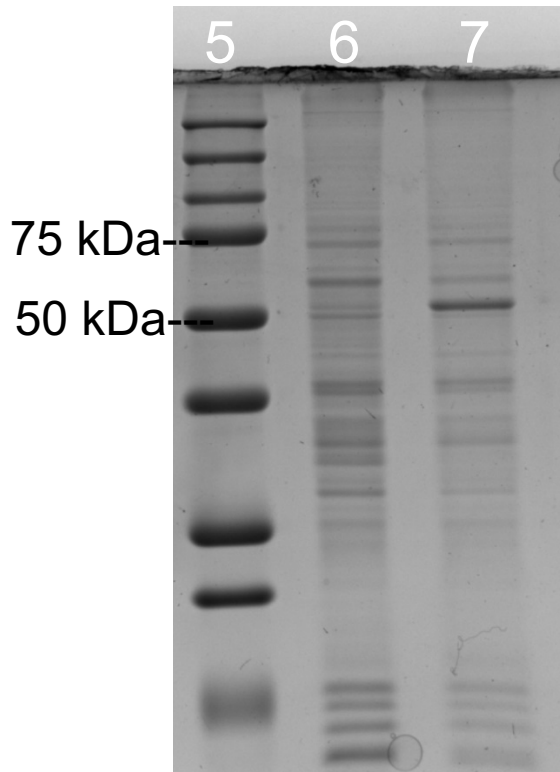

Supplement: S1 Fig — Lane 1: Precision Plus Protein ladder (Bio-rad). Lane 2: empty vector microsomes. Lane 3: CYP6BW1 microsomes, protein band is visible between 75 kDa and 50 kDa. Lane 4: CYP6BW3 microsomes, protein band is visible between 75 kDa and 50 kDa. Lane 5: Precision Plus Protein ladder (Bio-rad). Lane 6: empty vector microsomes. Lane 7: CYP6DJ1 microsomes, protein band is visible between 75 kDa and 50 kDa. (PDF) [file pone.0216753.s001.pdf]

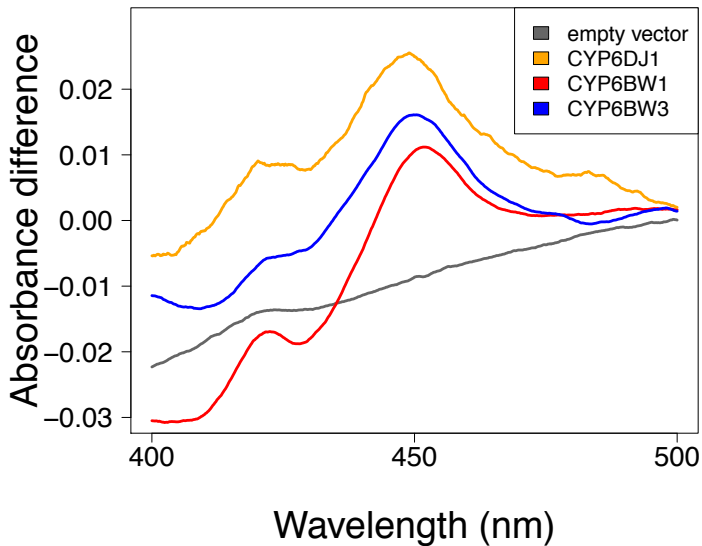

Supplement: S2 Fig — (PDF) [file pone.0216753.s002.pdf]

Ion Counts

Peak 3

Peak 4

Peak 5

Peak 6

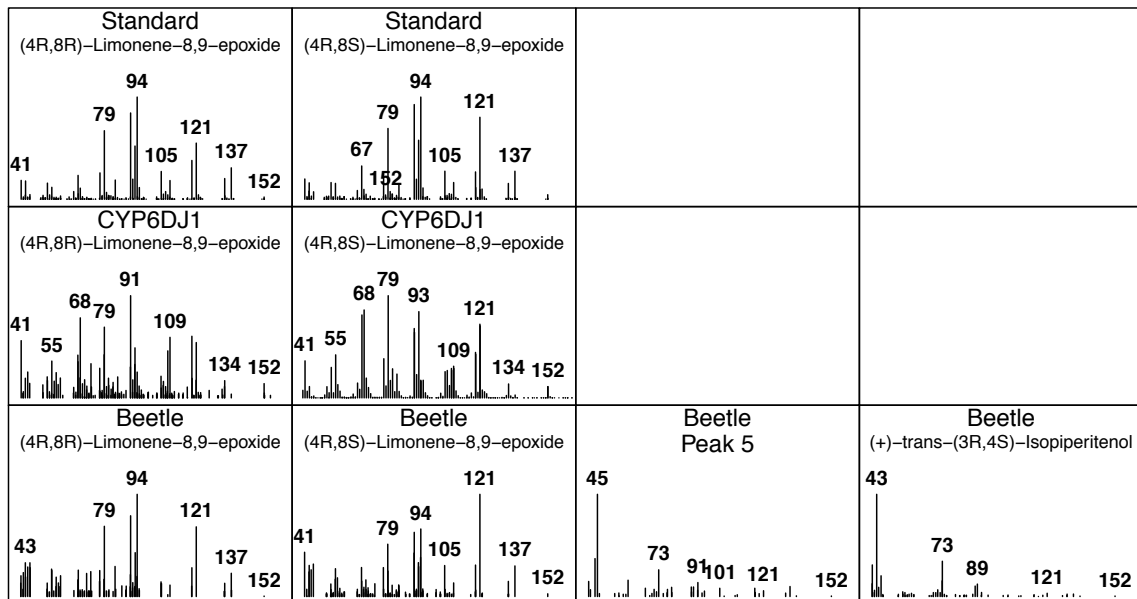

m/z

Peak 7

Peak 8

Peak 9

Ion Counts

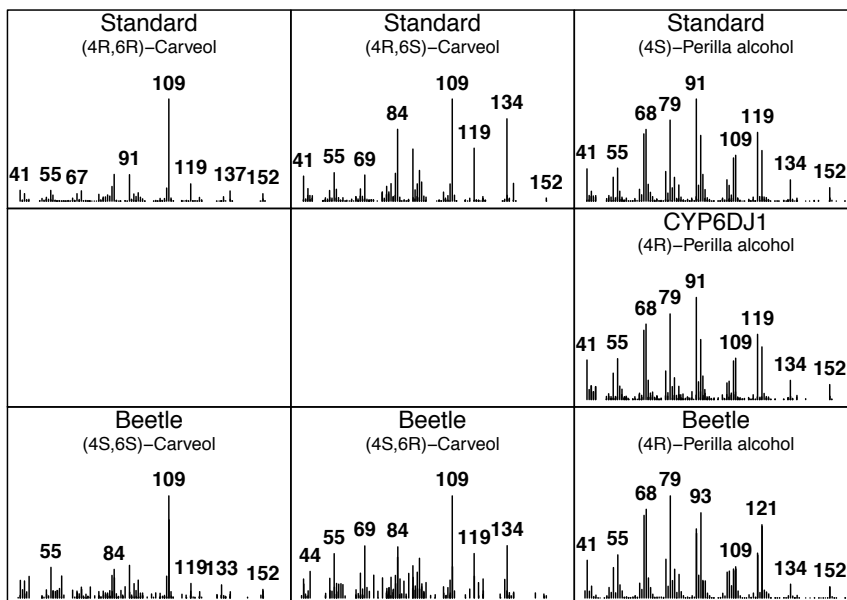

m/z

Supplement: S3 Fig — Gas chromatograms with peak numbers can be found in Fig 1. (PDF) [file pone.0216753.s003.pdf]

Ion Counts

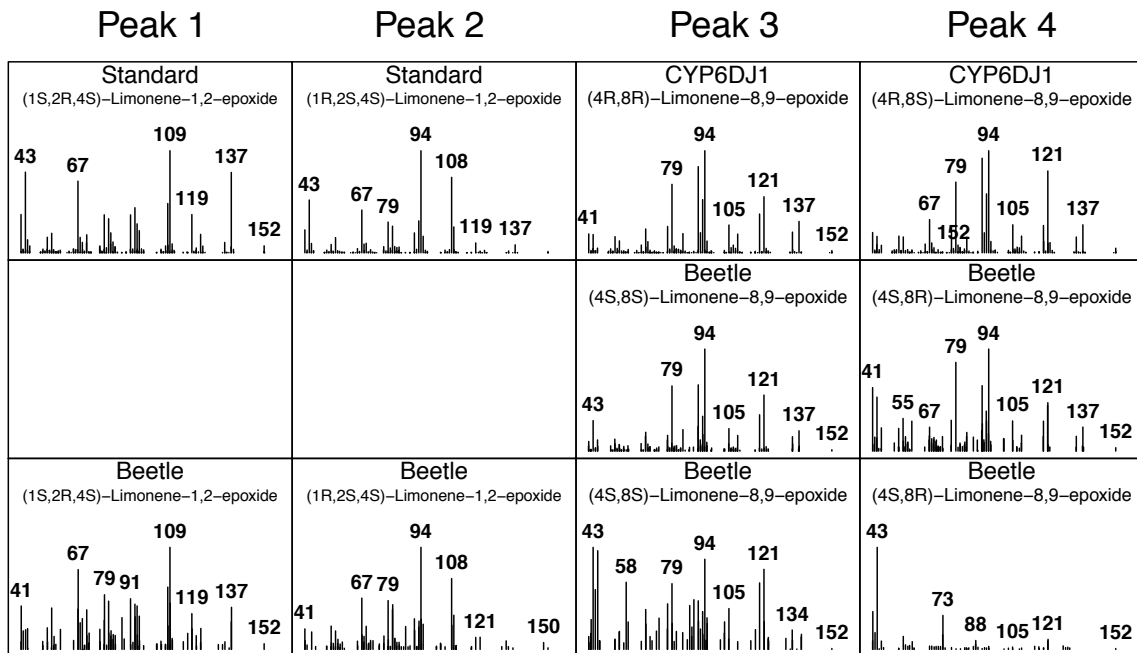

m/z

Ion Counts

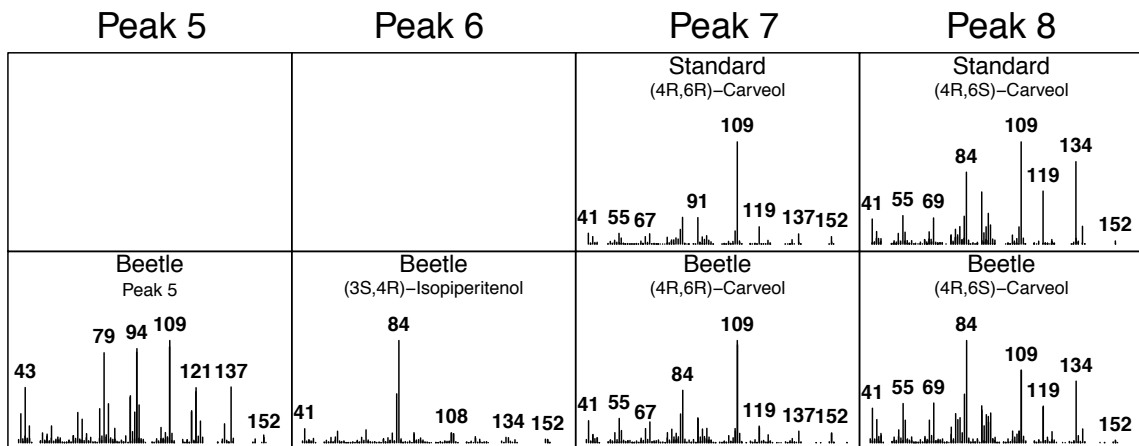

m/z

Supplement: S4 Fig — Gas chromatograms with peak numbers can be found in Fig 2. (PDF) [file pone.0216753.s004.pdf]

# Peak 9

# Peak 10

Ion Counts

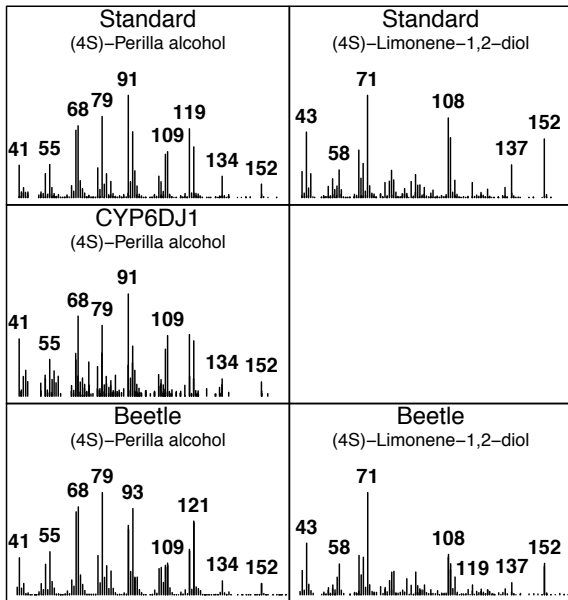

m/z

Supplement: S5 Fig — Gas chromatograms with peak numbers can be found in Fig 2. (PDF) [file pone.0216753.s005.pdf]

Peak 11

Peak 12

Peak 13

Peak 14

Ion Counts

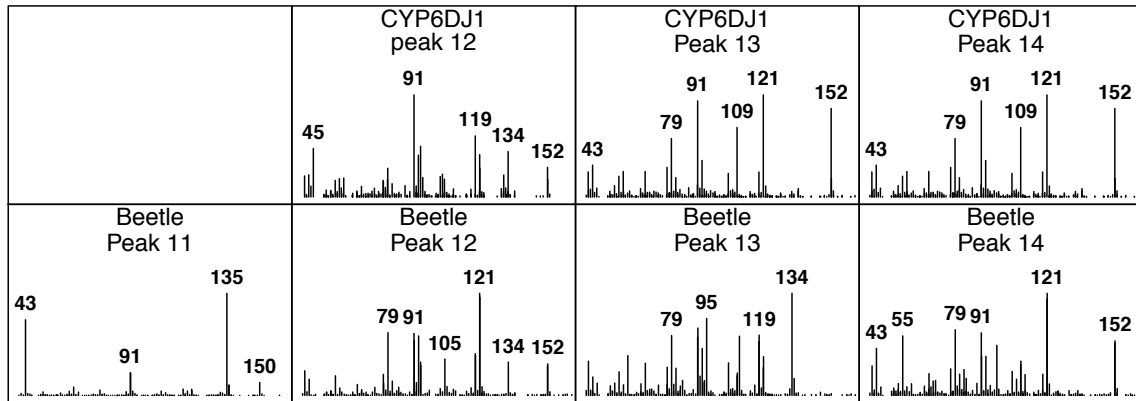 $m/z$

Supplement: S6 Fig — Gas chromatograms with peak numbers can be found in Fig 3. (PDF) [file pone.0216753.s006.pdf]

Ion Counts

Peak 19

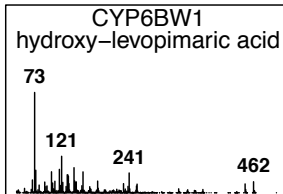

Peak 20

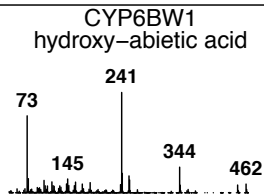

Peak 21

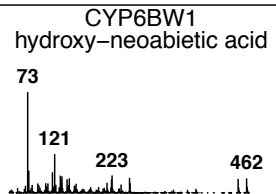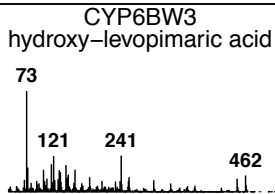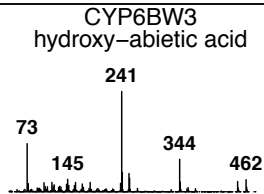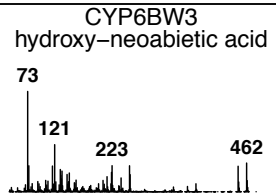

$m/z$

Supplement: S8 Fig — Gas chromatograms with peak numbers can be found in Fig 7D–7F. (PDF) [file pone.0216753.s008.pdf]
