# Supplementary material for: Functions of mountain pine beetle cytochromes P450 CYP6DJ1, CYP6BW1 and CYP6BW3 in the oxidation of pine monoterpenes and diterpene resin acids
Source: PLoS One. 2019 May 9;14(5):e0216753. doi: 10.1371/journal.pone.0216753 (PMC6508646; doi:10.1371/journal.pone.0216753)
Supplement: S7 Fig — Gas chromatograms with peak numbers can be found in Fig 7A–7C. (PDF) [file pone.0216753.s007.pdf]

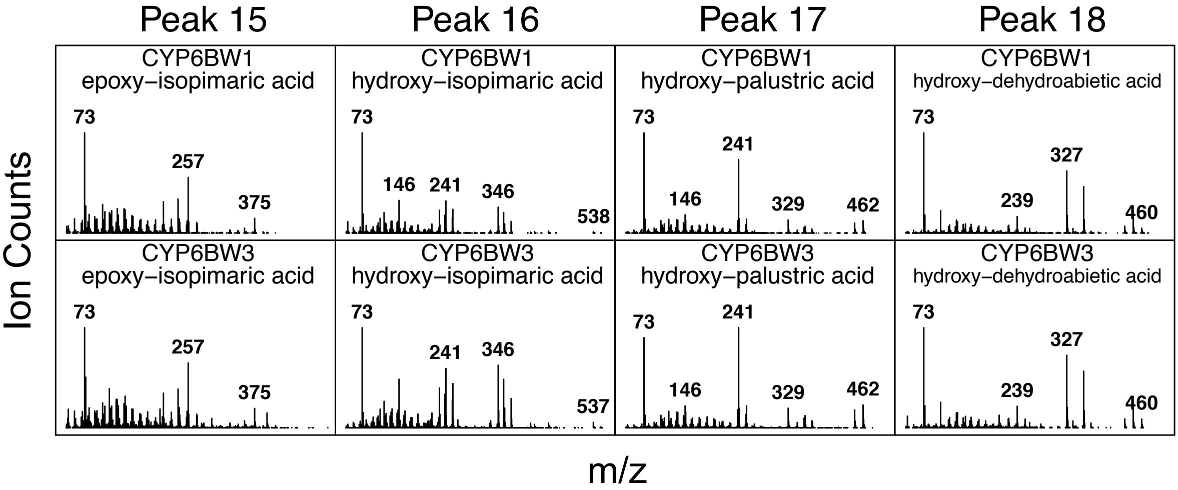
Peak 15
Peak 16
Peak 17
Peak 18
CYP6BW1
epoxy−isopimaric acid
73
257
375
CYP6BW1
hydroxy−isopimaric acid
73
146
241
346
538
CYP6BW1
hydroxy−palustric acid
73
241
146
329
462
CYP6BW1
hydroxy−dehydroabietic acid
73
327
239
460
CYP6BW3
epoxy−isopimaric acid
73
257
375
CYP6BW3
hydroxy−isopimaric acid
73
241
346
537
CYP6BW3
hydroxy−palustric acid
73
241
146
329
462
CYP6BW3
hydroxy−dehydroabietic acid
73
327
239
460
Ion Counts
m/z
