# Supplementary material for: Functions of mountain pine beetle cytochromes P450 CYP6DJ1, CYP6BW1 and CYP6BW3 in the oxidation of pine monoterpenes and diterpene resin acids
Source: PLoS One. 2019 May 9;14(5):e0216753. doi: 10.1371/journal.pone.0216753 (PMC6508646; doi:10.1371/journal.pone.0216753)
Supplement: S1 Table — Red crosses indicate that no product was detected in GC chromatograms of the assay, green checkmarks indicate that one or more products were detected in the assay. (PDF) [file pone.0216753.s009.pdf]

Substrate	Activity Assay (*in vitro*)		
**Monoterpenes**	CYP6DJ1	CYP6BW1	CYP6BW3
(+)-α-pinene	✗	✗	✗
(-)-α-pinene	✗	✗	✗
(+)-β-pinene	✗	✗	✗
(-)-β-pinene	✗	✗	✗
(+)-limonene	✓	✗	✗
(-)-limonene	✓	✗	✗
(+)-3-carene	✗	✗	✗
β-phellandrene	✗	✗	✗
myrcene	✗	✗	✗
terpinolene	✓	✗	✗
**Diterpene Resin Acids**			
abietic acid	✗	✓	✓
dehydroabietic acid	✗	✓	✓
neoabietic acid	✗	✓	✓
levopimaric acid	✗	✓	✓
palustric acid	✗	✓	✓
isopimaric acid	✗	✓	✓
