# Supplementary material for: Functions of mountain pine beetle cytochromes P450 CYP6DJ1, CYP6BW1 and CYP6BW3 in the oxidation of pine monoterpenes and diterpene resin acids
Source: PLoS One. 2019 May 9;14(5):e0216753. doi: 10.1371/journal.pone.0216753 (PMC6508646; doi:10.1371/journal.pone.0216753)
Supplement: S2 Table — All samples were injected onto a DB-Wax column. See Figs 1–5 and S3–S6 Figs. for the gas chromatograms, structures and mass spectra of these peaks. (PDF) [file pone.0216753.s010.pdf]

Reference	Retention Index	Compound name
Peak 1	1443	(1*S*,2*R*,4*S*)-limonene-1,2-epoxide
Peak 2	1456	(1*R*,2*S*,4*S*)-limonene-1,2-epoxide
Peak 3	1551	(4*R*,8*R*)-limonene-8,9-epoxide<br>(4*S*,8*S*)-limonene-8,9-epoxide
Peak 4	1556	(4*R*,8*S*)-limonene-8,9-epoxide<br>(4*S*,8*R*)-limonene-8,9-epoxide
Peak 5	1662	Unknown limonene product
Peak 6	1754	(+)-*trans*-(3*R*,4*S*)-isopiperitenol<br>(–)-*trans*-(3*S*,4*R*)-isopiperitenol
Peak 7	1832	(+)-*cis*-(4*S*,6*S*)-carveol<br>(–)-*cis*-(4*R*,6*R*)-carveol
Peak 8	1863	(+)-*trans*-(4*S*,6*R*)-carveol<br>(–)-*trans*-(4*R*,6*S*)-carveol
Peak 9	2008	(4*R*)-perilla alcohol<br>(4*S*)-perilla alcohol
Peak 10	2165	(4*S*)-limonene-1,2-diol
Peak 11	1773	Unknown terpinolene product
Peak 12	2032	Unknown terpinolene product
Peak 13	2040	Unknown terpinolene product
Peak 14	2094	Unknown terpinolene product
