# Supplementary material for: Functions of mountain pine beetle cytochromes P450 CYP6DJ1, CYP6BW1 and CYP6BW3 in the oxidation of pine monoterpenes and diterpene resin acids
Source: PLoS One. 2019 May 9;14(5):e0216753. doi: 10.1371/journal.pone.0216753 (PMC6508646; doi:10.1371/journal.pone.0216753)
Supplement: S3 Table — All samples were injected onto a HP-5 column. See Fig 7 and S7 and S8 Figs for the gas chromatograms and mass spectra of these peaks. (PDF) [file pone.0216753.s011.pdf]

Reference	Retention Index	Compound name
Peak 15	2655	epoxy-isopimaric product
Peak 16	2892	hydroxy-isopimaric product
Peak 17	2675	hydroxy-palustric product
Peak 18	2690	hydroxy-dehydroabietic product
Peak 19	2713	hydroxy-levopimaric product
Peak 20	2729	hydroxy-abietic product
Peak 21	2872	hydroxy-neoabietic product
